# Supplementary material for: Mlig-SKP1 Gene Is Required for Spermatogenesis in the Flatworm Macrostomum lignano
Source: Int J Mol Sci. 2022 Dec 1;23(23):15110. doi: 10.3390/ijms232315110 (PMC9740662; doi:10.3390/ijms232315110)
Supplement: Supplementary file 1 [file ijms-23-15110-s001.zip › ijms-2040011-supplementary.pdf]

Supplementary Materials file for «*Mlig-SKP1* gene is required for spermatogenesis in the flatworm *Macrostomum lignano*»

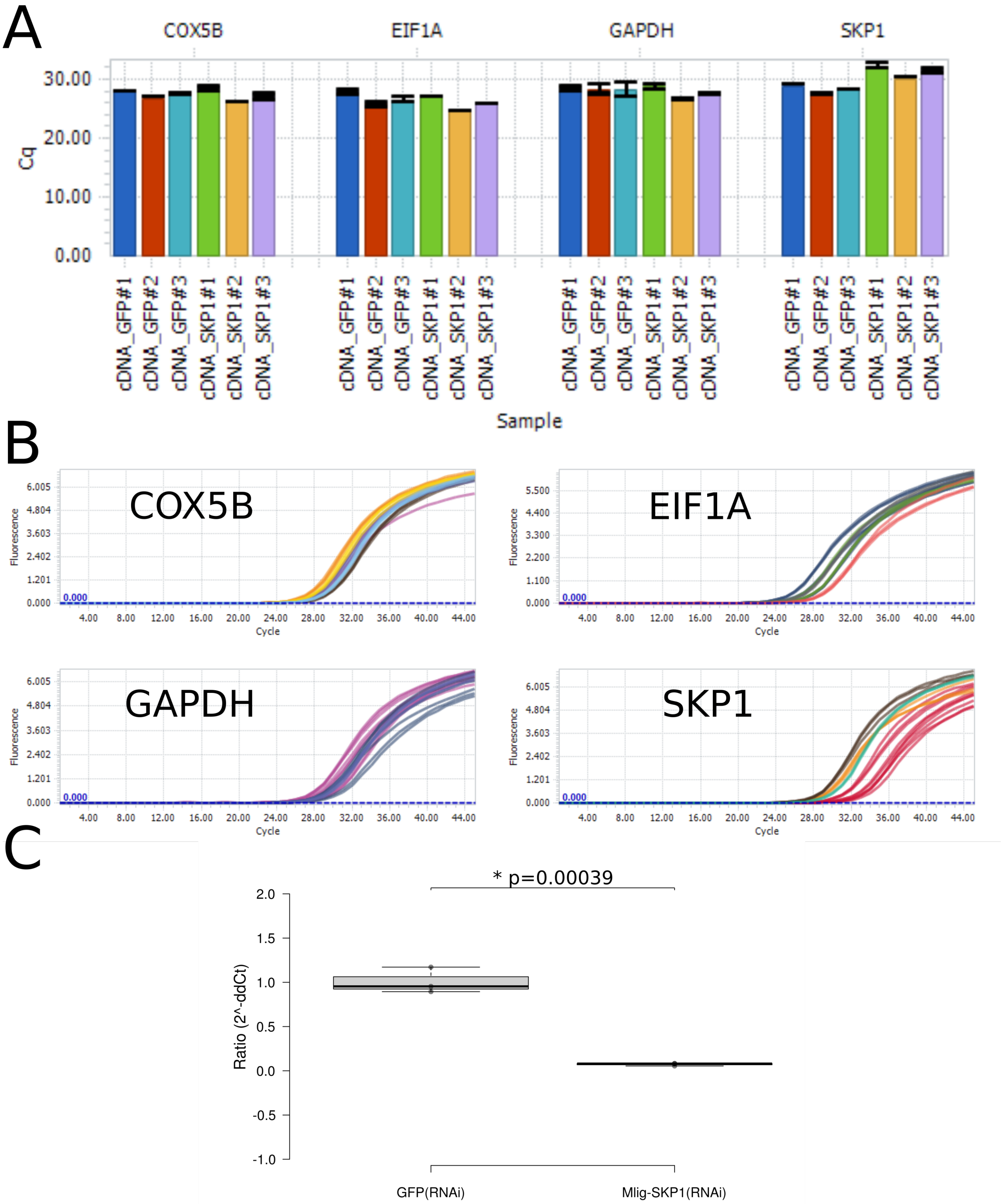

Figure S1. Verification of *Mlig-SKP1* knockdown by qRT-PCR. House-keeping genes *COX5B*, *GAPDH,* and *EIF1A* were used as controls. A - Bar-plots comparing raw *Mlig-SKP1* levels in knocked down worms and GFP knocked down worms as controls. For each of genes there are 3 repeats from different wells with worms treated with dsRNA against *GFP* or *Mlig-SKP1*. B - fluorescent curves for COX5B, EIF1A, GAPDH and SKP1 replicas; C - Decrease of *Mlig-SKP1* transcript levels as a result of knockdown estimated using ddCt method. The blue bar is a relative mRNA level in the control samples, the orange bar - in *Mlig-SKP1*(RNAi) samples.

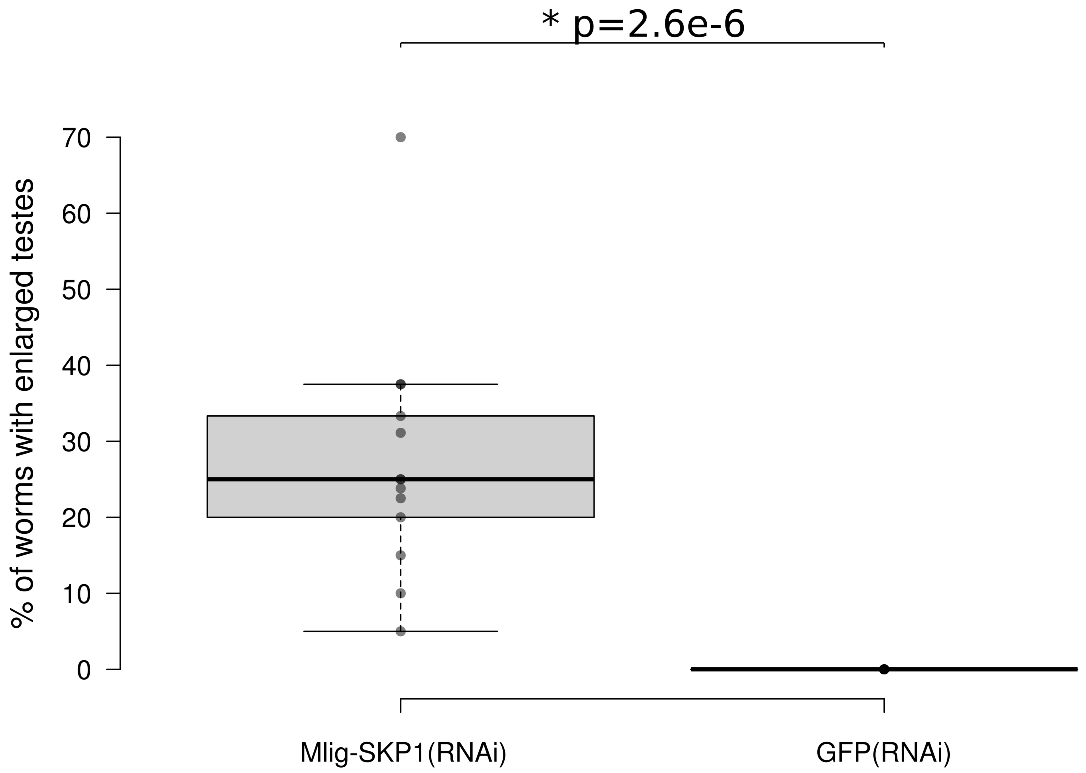

Figure S2. Number of worms with enlarged testes in *Mlig-SKP1*(RNAi) and *GFP*(RNAi) worms.

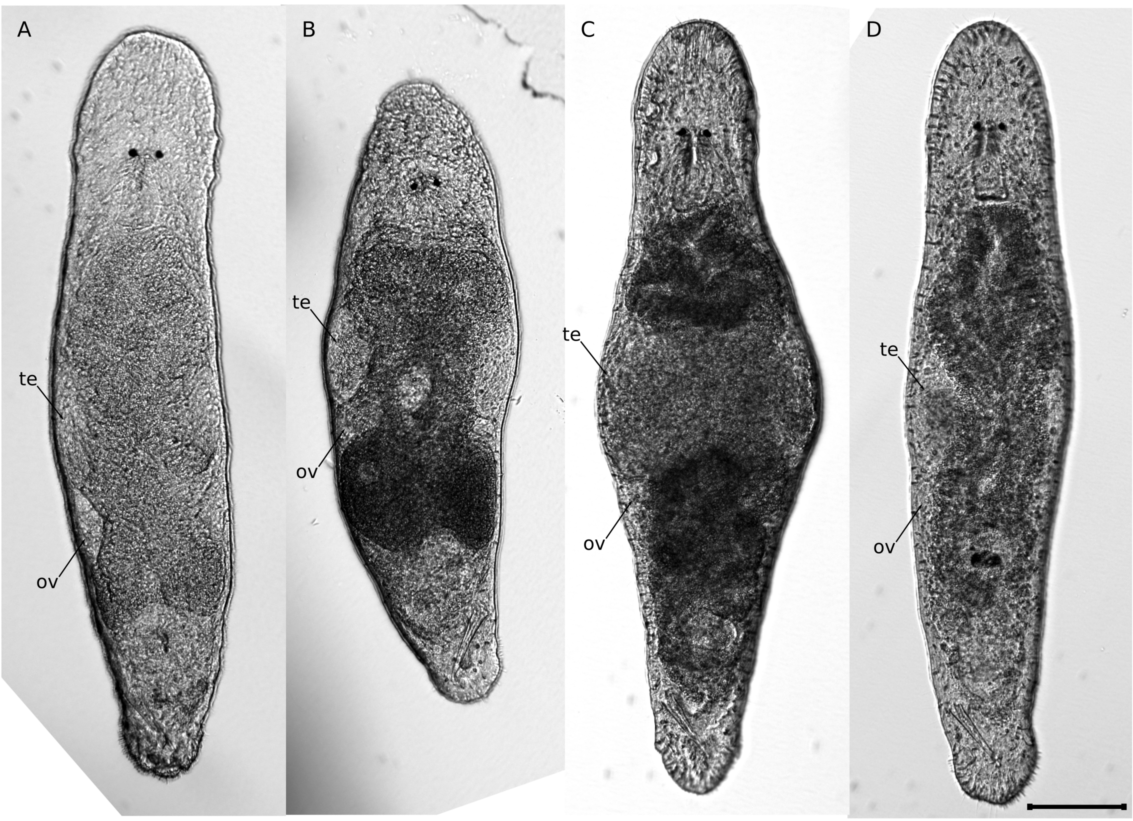

Figure S3. Variability of the testes size after *Mlig-SKP1* RNAi knockdown. A. A worm showing normal-sized testes. B. Small but abnormal testes become visually well-differentiated. C. A strong phenotype with enlarged testes. D. A phenotype after decreasing of the testes volume. Scale bar is 100 µm. Te-testes, ov – ovaries.

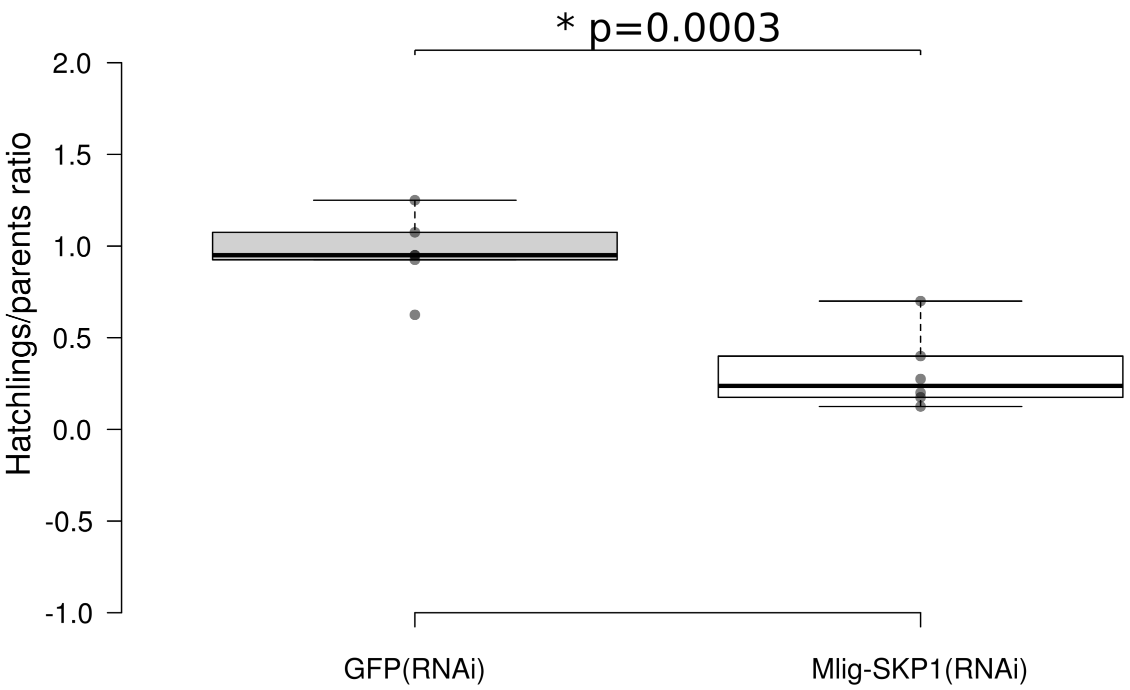

Figure S4. Effect of *Mlig-SKP1* RNAi knockdown on fertility in *M. lignano*.

Table S1. Primers used in the current project.

Primer Name	Primer Length (bp)	Seq (5' -> 3')	cDNA PCR product size (bp)
For cloning			
SKP1_RT-PCR_fwd	22	AGTCTCTGATGACTCCGATGAC	568
SKP1_RT-PCR_rev	20	AGAACAGCTGAGGCGATACA	
For qPCR			
SKP1_qPCR_fwd	21	CACAGCTATCCAACCAGAGAG	126
SKP1_qPCR_rev	19	GGTCCTCAAGCATGGTCTT	
COX5B_qPCR_fwd	22	CACAGTCACTATCGTCAGGTTG	95
COX5B_qPCR_rev	21	CCGCAGTAGCACCTTTGATTA	
EIF1A_qPCR_fwd	23	CGATGCCGAAGAATAAAGGAAAG	103
EIF1A_qPCR_rev	20	TCCTGACCATCCTCCTTGTA	
GAPDH_qPCR_fwd	20	GGGCGTCAATGAGGATTCTT	97
GAPDH_qPCR_rev	24	CGAATTCGTTGTTGATCACCTTAG	
